# Effectiveness of aromatase inhibitors and tamoxifen in reducing subsequent breast cancer

**DOI:** 10.1002/cam4.37

**Published:** 2012-09-26

**Authors:** Reina Haque, Syed A Ahmed, Alice Fisher, Chantal C Avila, Jiaxiao Shi, Amy Guo, T Craig Cheetham, Joanne E Schottinger

**Affiliations:** 1 Kaiser Permanente Southern CaliforniaPasadena, California, 91101; 2 Novartis Pharmaceuticals CorporationOne Health Plaza, East Hanover, New Jersey, 07936

**Keywords:** AI, breast cancer, comparative effectiveness, tamoxifen

## Abstract

Tamoxifen (TAM) has been prescribed for decades and aromatase inhibitors (AIs) have been used since the early 2000s in preventing subsequent breast cancer. However, outside of clinical trials, the effectiveness of AIs is not established. We examined the long-term risk of subsequent breast cancer among survivors treated with TAM and AIs in a large health plan. The study included 22,850 survivors, diagnosed with initial breast cancer (stages 0–IV) from 1996 to 2006, and followed 13 years maximum. We compared the risk of subsequent breast cancer in those who used TAM and/or AIs versus nonusers (the reference group). Hazard ratios (HR) adjusted for patient, tumor, treatment, and health-care characteristics were estimated using Cox models with time-dependent drug use status. Women who used TAM/AIs had a large reduction in risk of subsequent breast cancer compared with nonusers. While confidence intervals (CI) for all hormone treatment groups overlapped, women with high adherence (medication possession ratio ≥80%) who used AIs exclusively and had positive ER or PR receptor status had the greatest risk reduction (HR = 0.34, 95% CI: 0.28–0.41), followed by those who switched from TAM to AIs (HR = 0.39, 95% CI: 0.30–0.49), and those who used TAM exclusively (HR = 0.42, 95% CI: 0.36–0.47). Women with high adherence had the greatest risk reduction in subsequent breast cancer, but the results were not substantially different from women who took the drugs less regularly. Compared with nonusers, the reduction in subsequent breast cancer risk ranged from 58% to 66% across the hormone treatment groups and degree of adherence.

## Introduction

The American Cancer Society estimates that over 20,700 new cases of invasive breast cancer were expected in 2012, and that breast cancer will be responsible for one in 33 women's deaths [1, 2]. Next to radiation therapy, endocrine hormonal treatments, primarily tamoxifen (TAM) and aromatase inhibitors (AIs), are the most common adjuvant treatment for women with hormone receptor-positive breast cancer [3–7]. TAM acts by blocking estrogen binding to its receptor, while AIs block estrogen production [8]. It is generally recommended that women take these antiestrogen medications for 5 years (solely or sequentially), but duration of use varies in the general population. Earlier guidelines in the United States recommended AIs for treatment of late-stage breast cancer (stages III–IV) [4]; however, recent guidelines recommend use of AIs for all postmenopausal women diagnosed with invasive disease regardless of stage [8–10]. Guidelines also recommend that pre- or perimenopausal women at diagnosis take TAM for 5 years [11].

TAM has been prescribed for the past 25 years, and 20 mg/day is indicated for women with estrogen or progesterone-positive invasive breast cancer. Clinical trials found TAM decreases the risk of subsequent breast cancer by 50% [12]. However, data on its effectiveness in the general community are limited [13]. Additionally, while clinical trials are important for assessing treatment efficacy, such studies tend to include healthier women. AIs typically have been used following a period of TAM treatment; however, as a result of recent randomized clinical trials of AIs (letrozole, anastrozole, and exemestane) [14], current national guidelines recommend that women with hormone receptor-positive breast cancer take AIs as part of adjuvant treatment either up-front or following TAM [10]. A meta-analysis of clinical trials that evaluated AIs demonstrated a 48% reduction in contralateral breast cancer in comparison with TAM [15]. Nevertheless, while a number of clinical trials demonstrate the benefits of AIs [16–18], little is known about the extent of their effectiveness in diverse groups of breast cancer survivors in the community setting. Furthermore, the longest median follow-up in the recent clinical trials was 8 years, with the majority of studies having shorter follow-up. Notably, the number of events (subsequent breast cancer or deaths) was low in these trials, thus limiting inference. Therefore, our goal was to examine the long-term risk of subsequent breast cancer (up to 13 years of follow-up) in survivors of all stages of disease who were treated with TAM, AIs, or both using data from a large integrated health plan delivery system.

## Methods

### Data sources and setting

We conducted a cohort study of breast cancer survivors who were members of Kaiser Permanente Southern California (KPSC), a prepaid nonprofit integrated group practice health plan that serves over 3.2 million members. KPSC includes over 5000 physicians from multiple specialties who care for members at 15 medical centers and over 100 outpatient medical offices. Data elements for this study were extracted from electronic health records including membership, outpatient visits, diagnoses, procedures, hospital admissions and discharges, and pharmacy prescriptions. Virtually all members have pharmacy coverage. The study protocol was reviewed and approved by the KPSC Institutional Review Board.

### Patients and eligibility

Women over 18 years of age who had been members of the health plan for at least 1 year prior to breast cancer diagnosis and were diagnosed with their first breast cancer (stages 0–IV) between 1 January 1996 and 31 December 2006 were identified from the health plan's electronic SEER-affiliated (Surveillance Epidemiology and End Results) tumor registry. Women were included regardless of their ER/PR status. Women were excluded if they had a prior history of cancer (except nonmelanoma skin cancer), a bilateral diagnosis of breast cancer on the index diagnosis date, or if they lacked pharmacy benefits. We identified 25,577 women who met the eligibility criteria. We then excluded the following women: *n* = 758 due to long gaps in membership prior to index year, *n* = 1091 due to previous cancer, *n* = 207 who had bilateral breast cancer at index date, and *n* = 225 with missing stage information. We further excluded 446 women with nonstandard hormone treatment regimens (i.e., used AIs initially then switched to TAM or switched multiple times). This left a total of 22,850 eligible women for the analysis.

### Outcome definition

Subsequent breast cancer was defined to be invasive recurrences that occurred in the ipsilateral (same) breast, regional (e.g., lymph nodes in axilla, chest wall, or near clavicles), or distant sites. Because adjuvant hormonal treatment has the ability to reduce risk of recurrence in the same breast by 50% and has been equally effective in reducing risk in the contralateral breast, we examined the outcome (ipsilateral and contralateral events) as one dichotomous outcome (absence/presence) [19, 20]. Second primary (contralateral) breast cancer was identified from the SEER-affiliated tumor registry, while other recurrences were identified from electronic outpatient and inpatient records using *International Classification of Diseases Ninth Revision* (*ICD9*) diagnosis code 174 (malignant neoplasm female breast) or *ICD9* code 233 (ductal carcinoma in situ, DCIS) occurring more than 180 days after the index diagnosis date. These diagnoses had to be accompanied by a hospitalization or procedure code (biopsy, mastectomy, lumpectomy or other surgery, radiotherapy, chemotherapy) that occurred within 90 days following this later diagnosis. In women initially diagnosed with stage IV disease, we identified cancer progression using *ICD9* code 174 accompanied with radiology imaging, chemotherapy, or surgical procedure codes, although some of these procedures could have been done for palliative care. Additionally, we identified new tumors in other organs using the tumor registry.

### Cancer treatment and medication data

Information on primary cancer therapy (surgery, radiotherapy, and chemotherapy) was extracted from the SEER-affiliated tumor registry. We used computerized pharmacy data to identify filled TAM and AI prescriptions, dispensing dates, and days supplied after the index breast cancer diagnosis date. We estimated the total duration of each hormonal medication by summing the total days supplied for all prescriptions. The AIs that we examined were letrozole, anastrozole, and exemestane. The medication assessment period started on the earliest prescription dispensing date after the index breast cancer diagnosis and ended at one of the study endpoints (date of subsequent breast cancer diagnosis, death, health plan membership termination, or study's end). Women were classified into one of four exposure categories based on their hormone medication use patterns: (1) TAM only, (2) AI only, (3) switchers, and (4) nonusers of hormone treatment (the reference group). Women were categorized as “switchers” if they used TAM for at least 6 months and then used AIs for at least 6 months. Women who never used adjuvant hormonal treatment, or who used the medication for less than 6 months, were included in the reference group (labeled “nonusers” hence forward).

### Follow-up

We followed women until the date of subsequent breast cancer diagnosis, death, termination of health plan membership, or 31 December 2008, whichever occurred first. Thus, women were followed a maximum of 13 years (median 6.5 years, range 0.5–13 years). Dates of death were ascertained by linkage with electronic data from the State of California's master file of death certificates, electronic inpatient records, and the Social Security Administration file. Gaps of ≤60 days in enrollment were ignored as these were likely administrative gaps.

### Statistical analysis and covariates

We compared subsequent breast cancer risk with adjuvant hormonal treatment groups. Information on covariates specific to demographics, tumor characteristics, and cancer treatments was extracted from electronic health records and the SEER-affiliated tumor registry. Comorbidity status in the year prior to breast cancer diagnosis was determined using the Deyo adaptation of the Charlson Index [21]. Chi-square tests and *P*-values (two-sided) were used to examine the associations of demographics, primary and adjuvant cancer treatments, health-care utilization, and tumor characteristics with hormone medication use. Adjusted hazard ratios (HR) for subsequent breast cancer and 95% confidence intervals (CI) were estimated using Cox proportional hazards models with time-dependent drug use status. Hence, the person-time accrual began on the date of the hormonal treatment initiation [22]. The multivariable models were adjusted for age at diagnosis, diagnosis year, stage at diagnosis, menopausal status, race/ethnicity, comorbidity score, geocoded median household income, tumor size, histology, lymph node involvement, estrogen, progesterone and HER2/neu receptor status, health-care resource utilization, bisphosphonate use, first course of treatment (surgical, radiotherapy, chemotherapy), and an age with comorbidity interaction term.

In addition, we repeated the Cox multivariable analyses on a subset of women who had ≥80% medication possession ratio (MPR), a standard measure for estimating medication compliance [23]. The MPR was estimated as the number of days supply (excluding last refill) divided by the number of days between first and last dispense date. The 80% MPR is a recognized level that suggests that there are very few days without drugs on hand, and consequently fairly continuous medication usage.

## Results

As a part of their adjuvant treatment, 12.9% (*n* = 2939) used AIs exclusively, 11.1% (*n* = 2542) used both TAM and AIs (i.e., switchers), 29.7% (*n* = 6797) used TAM only, and 46.3% (*n* = 10,572) did not use either hormonal drug (Table 1). Baseline demographic characteristics are shown in Table 1. Use of AIs steadily rose in the early 2000s, with a marked increase occurring in 2003 [24]. Race/ethnicity was somewhat associated with type of hormonal treatment. Among the 2959 black women in the study, a greater fraction (56% or 1656/2959) did not use hormonal treatment compared with women of other backgrounds (*P* < 0.0001). Women in categories associated with higher geocoded median household income were more likely to use hormone therapy than women in lower income categories (*P* < 0.0001).

**Table 1:** Demographic characteristics of breast cancer survivors diagnosed 1996–2006

	Hormone treatment exposure at baseline diagnosis				
	
	Tamoxifen only	AI only	Both TAM and AI	No hormones	Total
Total	6797 (29.7)	2939 (12.9)	2542 (11.1)	10,572 (46.3)	22,850 (100.0)
Age at diagnosis (years)					
<40	328 (29.5)	24 (2.2)	68 (6.1)	690 (62.2)	1110 (4.9)
40–49	1321 (31.8)	184 (4.4)	418 (10.1)	2233 (53.7)	4156 (18.2)
50–59	1737 (26.9)	859 (13.3)	830 (12.9)	3020 (46.9)	6446 (28.2)
60–69	1698 (28.2)	1065 (17.7)	778 (12.9)	2480 (41.2)	6021 (26.4)
70–79	1243 (33.5)	579 (15.6)	368 (9.9)	1517 (40.9)	3707 (16.2)
80+	470 (33.3)	228 (16.2)	80 (5.7)	632 (44.8)	1410 (6.2)
					*P* < 0.0001
Year of diagnosis					
1996–1998	2206 (43.5)	66 (1.3)	474 (9.3)	2327 (45.9)	5073 (22.2)
1999–2001	2230 (35.8)	190 (3.1)	1158 (18.6)	2644 (42.5)	6222 (27.2)
2002–2004	1414 (21.0)	1237 (18.4)	811 (12.0)	3271 (48.6)	6733 (29.5)
2005–2006	947 (19.6)	1446 (30.0)	99 (2.1)	2330 (48.3)	4822 (21.1)
					*P* < 0.0001
Menopausal status					
Premenopausal	2527 (29.9)	542 (6.4)	869 (10.3)	4513 (42.7)	8451 (37.0)
Postmenopausal	4270 (29.7)	2397 (16.7)	1673 (65.8)	6059 (57.3)	14,399 (63.0)
					*P* < 0.0001
Race					
Non-Hispanic White	4551 (30.7)	1967 (13.3)	1743 (11.8)	6555 (44.2)	14,816 (64.8)
Hispanic	808 (28.8)	355 (12.7)	283 (10.1)	1360 (48.5)	2806 (12.3)
Black	701 (23.7)	335 (11.3)	267 (9.0)	1656 (56.0)	2959 (12.9)
Asian/Pacific Islander	699 (32.9)	257 (12.1)	244 (11.5)	927 (43.6)	2127 (9.3)
Other/Unknown*	38 (26.8)	25 (17.6)	5 (3.5)	74 (52.1)	142 (0.6)
					*P* < 0.0001
Geocoded median household income					
Lower 25% ($44,135 or lower)	1606 (29.1)	680 (12.3)	544 (9.9)	2688 (48.7)	5518 (24.1)
>25–50% (>$44,135–$59,275)	1663 (30.0)	667 (12.0)	606 (10.9)	2611 (47.1)	5547 (24.3)
>50–75% (>$59,275–$79,489)	1673 (30.2)	747 (13.5)	654 (11.8)	2473 (44.6)	5547 (24.3)
Top 25% ($79,490 or higher)	1622 (29.2)	778 (14.0)	692 (12.5)	2463 (44.3)	5555 (24.3)
Unknown/Missing*	233 (34.1)	67 (9.8)	46 (6.7)	337 (49.3)	683 (3.0)
					*P* < 0.0001

* Missing/unknown values were excluded in the estimation of *P*-value.

We also examined the distribution of health-care factors by use of hormonal treatments in the year before breast cancer diagnosis. The distribution of outpatient visits and hospitalizations was generally similar across hormonal drug users and nonusers (data not shown). The majority of the cohort had no comorbidities (78.4%, *n* = 17,920) in the year prior to diagnosis, while a small fraction had a Charlson score of ≥3 (3.6%, *n* = 817). Women who were switchers were less likely to have a high comorbidity score (≥3) compared with women from other categories (*P* < 0.0001). Regarding menopausal status, about 37.0% (*n* = 8451) of the cohort was postmenopausal. Within this group, 3938 women had used hormonal medications (2527 [29.9%] used TAM only; 542 [6.4%] used AIs solely; and 869 [10.3%] had used both).

Table 2 presents the distribution of tumor characteristics and primary and adjuvant treatments of the baseline breast cancer diagnosis by hormone drug use status. The majority of the cohort was diagnosed with early-stage disease (stages 0–II, 89.2%, *n* = 20,392). These results identify a potential small degree of off-label use of AIs among stage 0 survivors. Specifically, we found that among 3986 women with stage 0 disease (ductal carcinoma in situ), roughly 898 (22.5%) women used TAM while 52 (1.3%) used AIs and 27 (0.7%) used both following their baseline diagnosis. In addition, 1190 women initially diagnosed with stages III–IV disease used hormonal treatment following their late diagnosis. Of these, 744 used AIs solely or switched from TAM to AIs. Furthermore, a number of women with ER− tumors used TAM or AIs (*n* = 431). Of the 431 with ER− disease, 281 (65%) were PR+ (data not shown). Of note, the percentage of subsequent breast cancer in ER− women was the same regardless of whether they used hormone treatment (13% and 14%, respectively, data not shown).

**Table 2:** Tumor characteristics and treatment variables of the original breast cancer diagnosis

	Hormone treatment exposure with index diagnosis				
	
	Tamoxifen only	AI only	Both TAM and AI	No hormones	Total
Total	6797 (29.7)	2939 (12.9)	2542 (11.1)	10,572 (46.3)	22,850 (100.0)
Stages at diagnosis					
Stage 0	898 (22.5)	52 (1.3)	27 (0.7)	3009 (75.5)	3986 (17.4)
Stage I	3111 (35.9)	1345 (15.5)	923 (10.7)	3275 (37.8)	8654 (37.9)
Stage II	2342 (30.2)	1101 (14.2)	1289 (16.6)	3020 (39.0)	7752 (33.9)
Stage III	370 (20.6)	322 (18.0)	233 (13.0)	868 (48.4)	1793 (7.8)
Stage IV	76 (11.4)	119 (17.9)	70 (10.5)	400 (60.2)	665 (2.9)
					*P* < 0.0001
Primary therapy					
Breast-conserving surgery with radiation	2550 (34.6)	1038 (14.1)	923 (12.5)	2869 (38.9)	7380 (32.3)
Breast-conserving surgery (no radiation)	1336 (25.7)	561 (10.8)	393 (7.6)	2908 (55.9)	5198 (22.7)
Mastectomy (with or without radiation)	2784 (30.0)	1232 (13.3)	1162 (12.5)	4102 (44.2)	9280 (40.6)
No primary therapy	88 (10.9)	84 (10.4)	42 (5.2)	591 (73.4)	805 (3.5)
Other/Unknown/Missing*	39 (20.9)	24 (12.8)	22 (11.8)	102 (54.5)	187 (0.8)
					*P* < 0.0001
Chemotherapy					
Yes	2137 (24.8)	1124 (13.0)	1386 (16.1)	3967 (46.1)	8614 (37.7)
No	4402 (32.8)	1720 (12.8)	1014 (7.6)	6289 (46.8)	13,425 (58.8)
Unknown/Missing*	258 (31.8)	95 (11.7)	142 (17.5)	316 (39.0)	811 (3.5)
					*P* < 0.0001
Histology					
DCIS	382 (22.7)	23 (1.4)	7 (0.4)	1269 (75.5)	1681 (7.4)
LCIS (lobular carcinoma in situ)	52 (16.1)	2 (0.6)	2 (0.6)	266 (82.6)	322 (1.4)
IDC (invasive ductal carcinoma)	3681 (30.8)	1661 (13.9)	1443 (12.1)	5167 (43.2)	11,952 (52.3)
ILC (invasive lobular carcinoma)	493 (36.9)	253 (18.9)	254 (19.0)	336 (25.1)	1336 (5.8)
Other/Mixed category	2189 (29.0)	1000 (13.2)	836 (11.1)	3534 (46.8)	7559 (33.1)
					*P* < 0.0001
Grade					
1	1574 (36.6)	769 (17.9)	614 (14.3)	1341 (31.2)	4298 (18.8)
2	3085 (34.5)	1404 (15.7)	1175 (13.1)	3279 (36.7)	8943 (39.1)
3	1579 (20.9)	649 (8.6)	592 (7.8)	4739 (62.7)	7559 (33.1)
Unknown/Missing*	559 (27.3)	117 (5.7)	161 (7.9)	1213 (59.2)	2050 (9.0)
					*P* < 0.0001
Size of tumor (cm)					
No mass	17 (21.0)	12 (14.8)	7 (8.6)	45 (55.6)	81 (0.4)
<1	1264 (28.9)	510 (11.7)	314 (7.2)	2284 (52.2)	4372 (19.1)
1.0–1.9	2523 (34.8)	1144 (15.8)	884 (12.2)	2698 (37.2)	7249 (31.7)
2.0–2.9	1364 (30.0)	663 (14.6)	614 (13.5)	1899 (41.8)	4540 (19.9)
3.0–3.9	511 (25.9)	245 (12.4)	276 (14.0)	942 (47.7)	1974 (8.6)
4.0–4.9	224 (24.0)	115 (12.3)	117 (12.5)	478 (51.2)	934 (4.1)
5.0–9.9	286 (22.6)	139 (11.0)	163 (12.9)	679 (53.6)	1267 (5.5)
10+	26 (14.7)	19 (10.7)	15 (8.5)	117 (66.1)	177 (0.8)
Other/Unknown/Missing*	582 (25.8)	92 (4.1)	152 (6.7)	1430 (63.4)	2256 (9.9)
					*P* < 0.0001
Lymph nodes					
Positive	1690 (27.6)	1015 (16.6)	1150 (18.8)	2261 (37.0)	6116 (26.8)
Negative	3832 (32.8)	1681 (14.4)	1241 (10.6)	4916 (42.1)	11,670 (51.1)
Other/Unknown/Missing*	1275 (25.2)	243 (4.8)	151 (3.0)	3395 (67.0)	5064 (22.2)
					*P* < 0.0001
Estrogen receptor					
Positive	5441 (38.0)	2773 (19.4)	2305 (16.1)	3805 (26.6)	14,324 (62.7)
Negative	311 (7.4)	56 (1.3)	64 (1.5)	3787 (89.8)	4218 (18.5)
Other/Unknown/Missing*	525 (12.2)	79 (1.8)	126 (2.9)	1094 (25.4)	1824 (8.0)
Test not done	520 (12.1)	31 (0.7)	47 (1.1)	1886 (43.8)	2484 (10.9)
					*P* < 0.0001
Progesterone receptor					
Positive	3169 (37.7)	1616 (19.2)	1268 (15.1)	2360 (28.1)	8413 (36.8)
Negative	587 (11.7)	388 (7.7)	229 (4.6)	3820 (76.0)	5024 (22.0)
Other/Unknown/Missing*	869 (9.2)	244 (2.6)	317 (3.4)	1366 (14.5)	2796 (12.2)
Test not done	2172 (23.1)	691 (7.3)	728 (7.7)	3026 (32.1)	6617 (29.0)
					*P* < 0.0001
HER2/neu					
Positive	185 (15.3)	237 (19.6)	76 (6.3)	711 (58.8)	1209 (5.3)
Negative	1372 (22.5)	1516 (24.8)	750 (12.3)	2465 (40.4)	6103 (26.7)
Other/Unknown/Missing*	3501 (22.5)	761 (4.9)	1008 (6.5)	4773 (30.7)	10,043 (44.0)
Test not done	1739 (11.2)	425 (2.7)	708 (4.6)	2623 (16.9)	5495 (24.0)
					*P* < 0.0001

* Missing/unknown values were excluded in the estimation of *P*-value.

Hormone medication use varied by primary therapy (surgery with or without radiation) and also by adjuvant chemotherapy (*P* < 0.0001 for both variables) (Table 2). Among the 8614 women who underwent chemotherapy, nearly 46.1% (*n* = 3967) did not receive hormonal treatment. Women with invasive ductal carcinoma, mixed histology, and grade 2 lesions were more likely to use hormonal medications. The majority of women in this cohort (*n* = 20,326) had lesions of <1 cm in size. Within this group, 6171 women used TAM solely while 5182 women used AIs alone or following TAM. Among the 6116 women with positive lymph nodes roughly, 35.4% (2165/6116) had used AIs only or were switchers while 27.6% (1690/6116) used TAM only. As expected, women with ER+ or PR+ or HER2− lesions were more likely to use AIs solely or following TAM compared with women with ER− or PR− or HER2+ lesions (*P* < 0.0001 for all variables). Strikingly, 24.1% (3237/13,412) of the women with ER+ invasive disease did not use hormonal drugs, or used them for less than 6 months, as a part of their long-term therapy. This association was also seen with the PR+ status.

Table 3 presents the overall and adjusted impact of AIs, TAM, and switching drugs on subsequent breast cancer. The overall subsequent breast cancer rate was 25.37/1000 person-years (95% CI: 24.46–26.31 per 1000 person-years). The highest subsequent breast cancer rate was seen among women who did not use any hormonal drugs (34.25/1000 person-years). The top half of Table 3 displays the overall and adjusted hazard ratios among the whole cohort, while the bottom half presents the association among women who had better drug adherence as defined by having a ≥80% MPR. Among women with ≥80% MPR with TAM and AIs, adjusted HRs demonstrate that all users of hormone treatment had a strong reduction in subsequent lesions, from 46% to 60% lower compared with nonusers (the reference group). While confidence intervals for all hormone treatment groups overlapped among those with good drug adherence, women who used AIs exclusively had the greatest risk reduction (HR = 0.40, 95% CI: 0.33–0.48), followed by those who switched from TAM to AIs (HR = 0.47, 95% CI: 0.38–0.59), and those who used TAM exclusively (HR = 0.49, 95% CI: 0.43–0.54).

**Table 3:** Rate of subsequent breast cancer among survivors diagnosed between 1996 and 2006 and followed through 2008 by adjuvant hormonal treatment groups

Type	*N*	Person-years of exposure	Number of subsequent BCa	Rate per 1000 P-Y (95% CI)	Crude HR	95% CI	Adjusted HR^1^	95% CI
All women	22,850	114,892	2915	25.37 (24.46–26.31)				
Tamoxifen only	6797	45,987	807	17.55 (16.36–18.80)	0.50	0.46–0.55	0.54	0.49–0.60
AI only	2939	9440	230	24.37 (21.32–27.73)	0.62	0.54–0.72	0.45	0.39–0.53
Switchers^2^	2542	8422	130	15.44 (12.90–18.33)	0.64	0.53–0.77	0.49	0.40–0.60
No hormones	10,572	51,044	1748	34.25 (32.66–35.89)	1.00	Reference	1.00	Reference

Women with MPR^3^ ≥80%	19,105	93,460	2526	27.03 (25.98–28.10)				
Tamoxifen only	4388	30,897	514	16.64 (15.23–18.14)	0.46	0.42–0.51	0.49	0.43–0.54
AI only	2188	7003	162	23.13 (19.71–26.98)	0.57	0.48–0.67	0.40	0.33–0.48
Switchers^2^	1957	6453	102	15.81 (12.89–19.19)	0.66	0.53–0.81	0.47	0.38–0.59
No hormones	10,572	49,107	1748	35.60 (33.95–37.30)	1.00	Reference	1.00	Reference

1 Adjusted for age at diagnosis, year of diagnosis, race/ethnicity, household income, health-care visits, hospitalizations, comorbidity, stage at diagnosis, primary treatment, chemotherapy, histology, grade, tumor size, lymph nodes, ER, PR, and HER2/neu status, menopause status.

2 Switched from tamoxifen to aromatase inhibitor.

3 MPR, medication possession ratio.

When we repeated the analyses on the 14,640 subset of women with known hormone receptor status (ER+ or PR+), the risk reduction was even greater in all three drug user groups (Table 4). Of the 14,640 survivors, 11,509 women had high adherence. Among the 11,509 women with high adherence, those who used AIs exclusively had the greatest risk reduction (HR = 0.34, 95% CI: 0.28–0.41), followed by those who switched from TAM to AIs (HR = 0.39, 95% CI: 0.30–0.49), and those who used TAM exclusively (HR = 0.42, 95% CI: 0.36–0.47).

**Table 4:** Rate of subsequent breast cancer among survivors diagnosed between 1996 and 2006 with ER+ or PR+ receptor status and followed through 2008 by adjuvant hormonal treatment groups

Type	*N*	Person-years of exposure	Number of subsequent BCa	Rate per 1000 P-Y (95% CI)	Crude HR	95% CI	Adjusted HR^1^	95% CI
All women	14,640	73,985	1759	23.78 (22.68–24.91)				
Tamoxifen only	5546	37,711	654	17.34 (16.04–18.72)	0.42	0.37–0.46	0.46	0.41–0.52
AI only	2791	8902	221	24.83 (21.66–28.32)	0.54	0.46–0.63	0.37	0.32–0.44
Switchers^2^	2340	7756	117	15.09 (12.48–18.08)	0.52	0.42–0.64	0.40	0.32–0.50
No hormones	3963	19,616	767	39.10 (36.38–41.97)	1.00	Reference	1.00	Reference

Women with MPR^3^ ≥80%	11,509	56,516	1435	25.39 (24.09–26.74)				
Tamoxifen only	3633	25,854	419	16.21 (14.69–17.83)	0.37	0.33–0.42	0.42	0.36–0.47
AI only	2092	6646	158	23.77 (20.21–27.78)	0.49	0.41–0.58	0.34	0.28–0.41
Switchers^2^	1821	6008	91	15.15 (12.19–18.60)	0.52	0.41–0.66	0.39	0.30–0.49
No hormones	3963	18,007	767	42.59 (39.63–45.72)	1.00	Reference	1.00	Reference

1 Adjusted for age at diagnosis, year of diagnosis, race/ethnicity, household income, health-care visits, hospitalizations, comorbidity, stage at diagnosis, primary treatment, chemotherapy, histology, grade, tumor size, lymph nodes, ER, PR, and HER2/neu status, menopause status.

2 Switched from tamoxifen to aromatase inhibitor.

3 MPR, medication possession ratio.

We also repeated the model on a subset on women who were diagnosed with invasive cancer (i.e., we excluded women initially diagnosed with DCIS and also those who later developed DCIS as an endpoint) who did not receive chemotherapy (*n* = 8720). In this subgroup of women with high medication adherence, the similar reduction in subsequent breast cancer was seen for each treatment group as those models that included women with DCIS (Table 5). Again, the protection was the greatest in women who used AIs exclusively (HR = 0.35, 95% CI: 0.28–0.43), followed by switchers (HR = 0.37, 95% CI: 0.27–0.50) and those who used TAM exclusively (HR = 0.40, 95% CI: 0.34–0.46). In a different contrast, when we removed women who did not receive any hormonal treatment, the impact of exclusive AI use (HR = 0.96, 95% CI: 0.72–1.29) was similar to TAM (HR = 1.00, reference group) further confirming that protection conferred by the two medications is similar in magnitude (Table 5).

**Table 5:** Rate of subsequent breast cancer among survivors diagnosed between 1996 and 2006 with ER+ or PR+ receptor status and followed through 2008 by adjuvant hormonal treatment groups among women with invasive disease and not exposed to chemotherapy

Type	*N*	Person-years of exposure	Number of subsequent BCa	Rate per 1000 P-Y (95% CI)	Crude HR	95% CI	Adjusted HR^1^	95% CI
Women with MPR^3^ ≥80%	8720	43,507	979	22.50 (21.11–23.96)				
Tamoxifen only	2964	21,196	314	14.81 (13.22–16.55)	0.37	0.32–0.43	0.40	0.34–0.46
AI only	1611	5083	118	23.22 (19.22–27.80)	0.51	0.41–0.62	0.35	0.28–0.43
Switchers^2^	1294	4308	60	13.93 (10.63–17.93)	0.50	0.38–0.67	0.37	0.27–0.50
No hormones	2851	12,921	487	37.69 (34.42–41.19)	1.00	Reference	1.00	Reference

Women with MPR^3^ ≥80%	5869	30,586	492	16.09 (14.70–17.57)				
Tamoxifen only	2964	21,196	314	14.81 (13.22–16.55)	1.00	Reference	1.00	Reference
AI only	1611	5083	118	23.22 (19.22–27.80)	1.47	1.18–1.83	0.96	0.72–1.29
Switchers^2^	1294	4308	60	13.93 (10.63–17.93)	1.06	0.79–1.41	0.87	0.64–1.19

1 Adjusted for age at diagnosis, year of diagnosis, race/ethnicity, household income, health-care visits, hospitalizations, comorbidity, stage at diagnosis, primary treatment, histology, grade, tumor size, lymph nodes, ER, PR, and HER2/neu status, menopause.

2 Switched from tamoxifen to aromatase inhibitor.

3 MPR, medication possession ratio.

**Figure 1:**
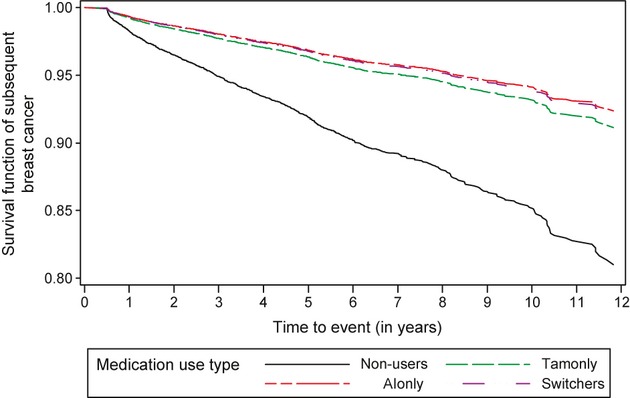
Adjusted survival functions for TAM-only users, AI-only users, switchers and nonusers of adjuvant hormonal therapy.

Figure 1 displays the adjusted survival functions for the three types of adjuvant treatment (TAM, AI, switchers) and nonusers controlling for other covariates as listed in Tables 3–5. The survival was highest in women who took AIs exclusively, but the curve for women who used TAM only was similarly high. The curve for the switchers overlapped with that of the AI exclusive users. Given that these curves are adjusted for the covariates as in the models, they demonstrate that the subsequent breast cancer rates were similar for the switchers as those who used AIs exclusively. Also, subsequent breast cancer rates begin to diverge after 5 years of follow-up in the AI and TAM groups, but not significantly.

## Discussion

This population-based study examined the long-term risk of subsequent breast cancer in a group of nearly 23,000 women treated with TAM, AIs, or both as compared with nonusers of hormonal treatment. There are several critical lessons gained from this population-based study. These data suggest that women who take AIs alone or following TAM treatment have subsequent breast cancer rates similar to women treated exclusively with TAM, and that all types of hormone treatment show markedly lower rates of subsequent breast cancer as compared with nonusers. This study also demonstrates the continued use of TAM as an important drug to reduce subsequent breast cancer risks as the rate of such lesions was significantly reduced over the 13-year follow-up period in this group. Furthermore, although the rate of subsequent breast cancer was lowest in women with high drug adherence, they were not substantially different from women who took the drugs less regularly. Hence, this study demonstrates the benefit of taking hormonal medications even if women take such drugs irregularly. This is a critical point and offers providers options for treating women. For example, women might be struggling physically (due to side effects) or financially while taking these medications daily, and if survivors are encouraged to continue their medications for 6 months or longer, but less than the recommended 5 years, their risk of subsequent breast cancer may still be reduced. Additionally, women over age 65 may have gaps in prescription usage due to the “doughnut hole” in Medicare coverage; however, this study supports the continued use of generic TAM as a treatment option [5, 20, 25].

In a large diverse community of breast cancer survivors, our results confirm the effectiveness of adjuvant hormonal therapy. The results of this study generally corroborate the findings of the randomized clinical trials of AIs that have examined subsequent breast cancer tumors as endpoints [9, 12, 14–18]. Although each trial examined slightly different outcomes and shorter follow-up as compared with this study, the rates of subsequent breast cancer events were lower in the groups that used AIs [26]. Compared with nonusers, women who solely used AIs had the greatest reduction in risk, but the magnitude of effect was similar in women who used both TAM and AIs.

This study offers insight into the diffusion of these medications in “real” world settings. Astonishingly, 24.1% of the women with invasive ER+ disease prescribed hormonal drugs did not use them as a part of their long-term therapy. Another recent study similarly determined that a large fraction of breast cancer survivors in another health plan did not use these treatments [27, 28]. As a result of these findings, our health plan will examine contraindications using medical records to identify postmenopausal women who should be targeted for such treatment. It is possible that such women had a history of peripheral thromboembolic disease or other contraindications. We also found a small degree of off-label use of hormone treatment among stage 0 (DCIS) survivors. Specifically, of the 3986 women with DCIS, 52 (1.3%) women used AIs and 27 (0.7%) used both AIs and TAM following their baseline diagnosis. We also found that a small fraction of women with ER− tumors used hormonal treatment (*n* = 431); however, a large fraction of these women were also PR+ (65%). It is unclear from the electronic data why these particular women used hormonal treatments, but it is possible that they may have had risk factors associated with subsequent breast cancer that we could not capture such as being a BRCA mutation carrier or family history. In addition, some women diagnosed with HER2-positive tumors prior to 2005 might have had higher rates of subsequent breast cancer because of the unavailability of trastuzumab, which was approved by the FDA in 2005.

Certain limitations of this study need to be considered. We were not able to examine why women switched from TAM to AIs. In addition, we may not have captured all subsequent breast cancer lesions through the electronic clinical records; hence, the actual protective effects of these adjuvant hormonal treatments may in fact be greater. The ideal source for capturing subsequent breast cancer lesions would have been the paper medical charts, but reviewing this large number was not feasible. Despite this limitation, the subsequent breast cancer rate in this study is consistent with previous chart review-based studies of other health plans in the United States with similar follow-up periods [19, 29]. Although we captured a comprehensive number of covariates, residual confounding is possible in this observational study.

This study has a number of advantages. Given its large sample, the diverse cohort included nearly 3000 black women and 2800 Hispanic women. Another advantage was our access to comprehensive pharmacy records with up to 13 years of follow-up. The study was based in an integrated health plan where patients receive virtually all their care within the system. The major reason for loss to follow-up was death (median length of membership was 20 years among the subjects including the pre- and post-breast cancer diagnosis periods; data not shown). The results of this study may not be generalizable to all settings as the study group included insured women; however, the characteristics of the KPSC membership are similar to the communities in which it serves in terms of race/ethnicity and income distribution.

Future studies of hormonal treatments should consider how potential side effects of the hormonal treatments impact discontinuation [30–32]. Additional long-term population-based studies are needed to determine whether risk of subsequent breast cancer is reduced by initially starting on an AI or by taking specific AIs following a certain number of years of TAM use, if particular AIs are more effective than others, and if combinations of hormonal treatments beyond the first 5 years improve survival.
